# Pre-expansion downward maxillary base rotation as a biomarker for post-expansion downward maxillary rotation in adults treated with 3D-guided midpalatal piezocorticotomy-assisted MARPE: a pilot case-control study

**DOI:** 10.3389/froh.2026.1900614

**Published:** 2026-08-10

**Authors:** Svitlana Koval, Daria Chepanova, Nika Stepanoff, Andrii Babii, Andrew Wellman

**Affiliations:** 1 DrKoval Orthodontics, Practice Limited to Orthodontics, Boca Raton, FL, United States; 2 Nova Southeastern University, Fort Lauderdale, FL, United States; 3 Division of Sleep and Circadian Disorders, Sleep Disordered Breathing Lab, Brigham and Women’s Hospital and Harvard Medical School, Boston, MA, United States

**Keywords:** 3D-guided piezocorticotomy, adults, downward maxillary rotation, MARPE, midpalatal suture

## Abstract

**Introduction:**

Structural predictors of success or failure in adult maxillary expansion remain poorly defined. This retrospective case-control pilot study aimed to evaluate the predictive capacity of pre-treatment craniofacial morphology—specifically the palatal plane inclination (SN-PP)—on final post-treatment maxillary skeletal morphology in adults undergoing 3D-Guided Midpalatal Piezocoticotomy Assisted Miniscrew-assisted rapid palatal expansion (3D-GMP MARPE).

**Methods:**

A total of eighty skeletally mature patients were evaluated, comprising a control group (mean age: 35.7 ± 8.4 years) and a case group (mean age: 33.1 ± 10.1 years) exhibiting sleep-disordered breathing and/or transverse maxillary deficiency underwent three-dimensional (3D) digitally guided midpalatal piezocoticotomy-assisted MARPE. Baseline metrics were derived from cone-beam computed tomography (CBCT) datasets in NemoFab software. Subjects were stratified based on their final absolute post-treatment morphology into cases (SN-PP >=11 degrees, *n* = 15) or controls (SN-PP < 11degrees, *n* = 65). Univariable and multivariable logistic regressions calculated crude and adjusted odds ratios (aOR) controlled for biological sex. Classification accuracy was evaluated using Receiver Operating Characteristic (ROC) curve analysis.

**Results:**

Baseline demographics, sagittal relationships, and soft-tissue thicknesses were statistically identical between groups (*p* > .05). Conversely, cases exhibited a significantly steeper baseline SN-PP_b angle than controls (11.5 ± 2.9° vs. 7.3 ± 3.1°, *p* = .001). Univariable regression revealed a 57% increase in the odds of case status per degree increase in SN-PP_b (OR=1.57; 95% CI: 1.23, 2.00; *p* < .001). This signal remained robustly significant after adjusting for sex in the multivariable model (aOR=1.51; 95% CI: 1.18, 1.94; *p* = .001). ROC analysis demonstrated excellent diagnostic discrimination (AUC = 0.841), backed by a *post-hoc* statistical power of 99.7%.

**Conclusions:**

A steeper pre-treatment palatal plane angle is a powerful, sex-independent predictor of a high-angle post-treatment skeletal relationship. Integrating SN-PP into diagnostic protocols provides high classification accuracy for pre-operative risk stratification regarding final skeletal phenotype.

## Introduction

Background. Rationale. Various Mini screw Assisted Rapid Palatal Expansion (MARPE) designs have shown high success rates in both adolescents (1) and adult patients (2, 3). Both direct and indirect positioning of the tooth-bone-borne midpalatal expansion appliances showed predictable midpalatal suture separation. Classifications were suggested based on the expansion efficiency and separation patterns (parallel/V-shaped) with non-piezocorticotomy MARPE showing that age is a reliable predictor for both efficiency and pattern of expansion (4). Historically, surgically assisted rapid palatal expansion (SARPE) has been considered a golden standard of skeletally achieved midpalatal separation in adults. Advanced expansion devices were described in conjunction with SARPE (5) showing higher levels of success and stability compared to conventional bonded hyrax expanders. Lately, several studies compared MARPE to SARPE in adult patients showing more parallel expansion patterns, higher levels of expansion in posterior palatal regions in MARPE patients (6).

Study by Ngan and Wilmes concluded less dento-alveolar effects of tooth-borne maxillary expander combined with the facemask over hybrid-hyrax combined with the facemask in growing subjects (7). This study is supported by findings reported by Demirsoy and colleagues (8) who reported equally effective hybrid-MSE and bone-borne expanders in young adults for correcting skeletal crossbites.

Sharma reported significantly greater increase in width measurements when comparing custom MARPE to conventional MARPE in young adult patients (9). Several minimal surgically-assisted techniques were described over the past five years including midpalatal corticotomies (10) and the MISMARPE approach (11) as auxiliaries to conventional MARPE approach. The corticotomy-assisted MARPE expansion was shown to reduce intra-expansion pain levels compared to non-assisted MARPE (10).

3D guided midpalatal piezocorticotomy (3D-GMP) assisted MARPE was introduced in 2025 after two years of clinical observations (12). The method is intended to increase the success rate of MARPE in adults and eliminate the age and sex parameters as limitations. Secondary outcomes of the 3D-GMP include achieving parallel and symmetrical separation of the midpalatal suture, preventing nasal septum from displacement in either direction while allowing for more predictable pterygomaxillary suture separation.

Despite the increasing success rate of MARPE in different age groups, the concern still exists regarding potential downward rotation of the maxillary complex and increase in the middle and lower anterior facial heights due to midfacial expansion.

## Objectives

Therefore, the primary objective of this retrospective case-control study was to identify baseline predictors of a high-angle post-treatment maxillary morphology (SN-PP >= 11) following maxillary 3D-GMP MARPE. Secondary objectives were to: (1) characterize baseline hard-tissue cephalometric variations and the two main areas of suture distraction (midpalatal suture and frontonasal suture) between individuals who developed the target condition (cases) and those who remained complication-free (controls), (2) determine whether the predictive signal of the primary skeletal biomarker persists independently when adjusting for baseline demographic distributions such as biological sex, and (3) quantify the definitive diagnostic and classification accuracy of this pre-treatment metric using receiver operating characteristic (ROC) curve analysis. We hypothesized that a steeper baseline palatal plane inclination would be significantly associated with case status, operating as an independent, sex-neutral structural risk biomarker with high diagnostic discrimination.

## Methods

### Study design

This study utilized a retrospective case-control design to evaluate pre-treatment craniofacial morphology as a predictor of post-expansion downward maxillary skeletal rotation. The study protocol was reviewed and approved by the Solutions IRB (protocol number 0486/1-13/2026), and was conducted in strict accordance with the Declaration of Helsinki. The written informed consent was obtained from all participants. This study was reported in accordance with the Strengthening the Reporting of Observational Studies in Epidemiology (STROBE) reporting guidelines.

A total of 80 subjects were consecutively selected from the electronic dental and radiographic databases of private practice DrKoval Orthodontics, Boca Raton, Florida, USA, between January and March 2026. Subjects were divided into two cohorts based on their clinical outcomes following standard pre-treatment diagnostic imaging: Case Group [Group 1 (*n* = 15)]: Defined as patients who developed downward maxillary rotation post-expansion and control Group [Group 2 (*n* = 65)]: Defined as patients who underwent identical procedure (3D-Guided Midpalatal Piezocorticotomy Assisted MARPE but remained free of post-expansion downward maxillary rotation.

### Intervention

All participants underwent a standardized, uniform first-stage treatment protocol designed to mechanically address structural transverse maxillary deficiency or deficit of the nasal cavity base. One hour prior to the surgical procedure, subjects received prophylactic oral pre-medication consisting of either 1000 mg of Amoxicillin or 300 mg of Clindamycin in cases of penicillin allergy, paired with an oral analgesic of either 800 mg of Ibuprofen or 1000 mg of Acetaminophen.

**Figure 1:**
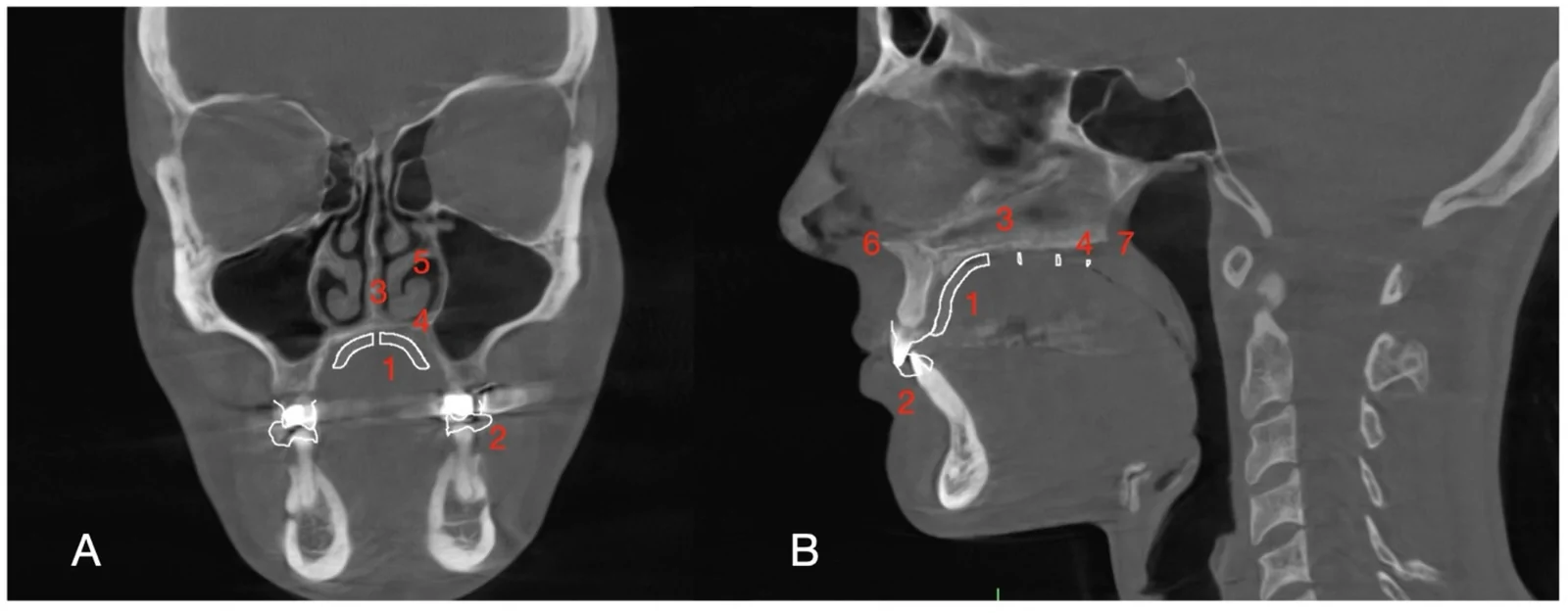
Digital design of the 3D midpalatal piezocorticotomy guide: 1- the baseplate of the guide, 2 – occlusal coverage of both the maxillary and mandibular incisors with the imprints of the mandibular teeth, 3 – nasal septum, 4 – palatal plane, 5 – inferior nasal turbinate, 6 – anterior nasal spine, 7 – posterior nasla spine.

The surgical intervention was performed strictly under local anesthesia. Topical anesthesia was established initially using 4% Cetacaine, followed by infiltrative local anesthesia utilizing either 3% lidocaine with 1:100,000 epinephrine or 0.5% bupivacaine (Marcaine) with 1:200,000 epinephrine. The three-dimensional (3D) surgical guide was custom-designed for each patient by digital fusion of individual pre-treatment CBCT datasets and intraoral scans (Figure 1). Using this digital template, a 3D-guided midpalatal piezocorticotomy was executed. The depth of the cortical incisions was customized to each subject, relying precisely on the anatomical thickness of the cortical bone surrounding the midpalatal suture. Horizontally, the surgical incision extended from the posterior aspect of the incisive foramen to the posterior nasal spine (PNS).

**Figure 2:**
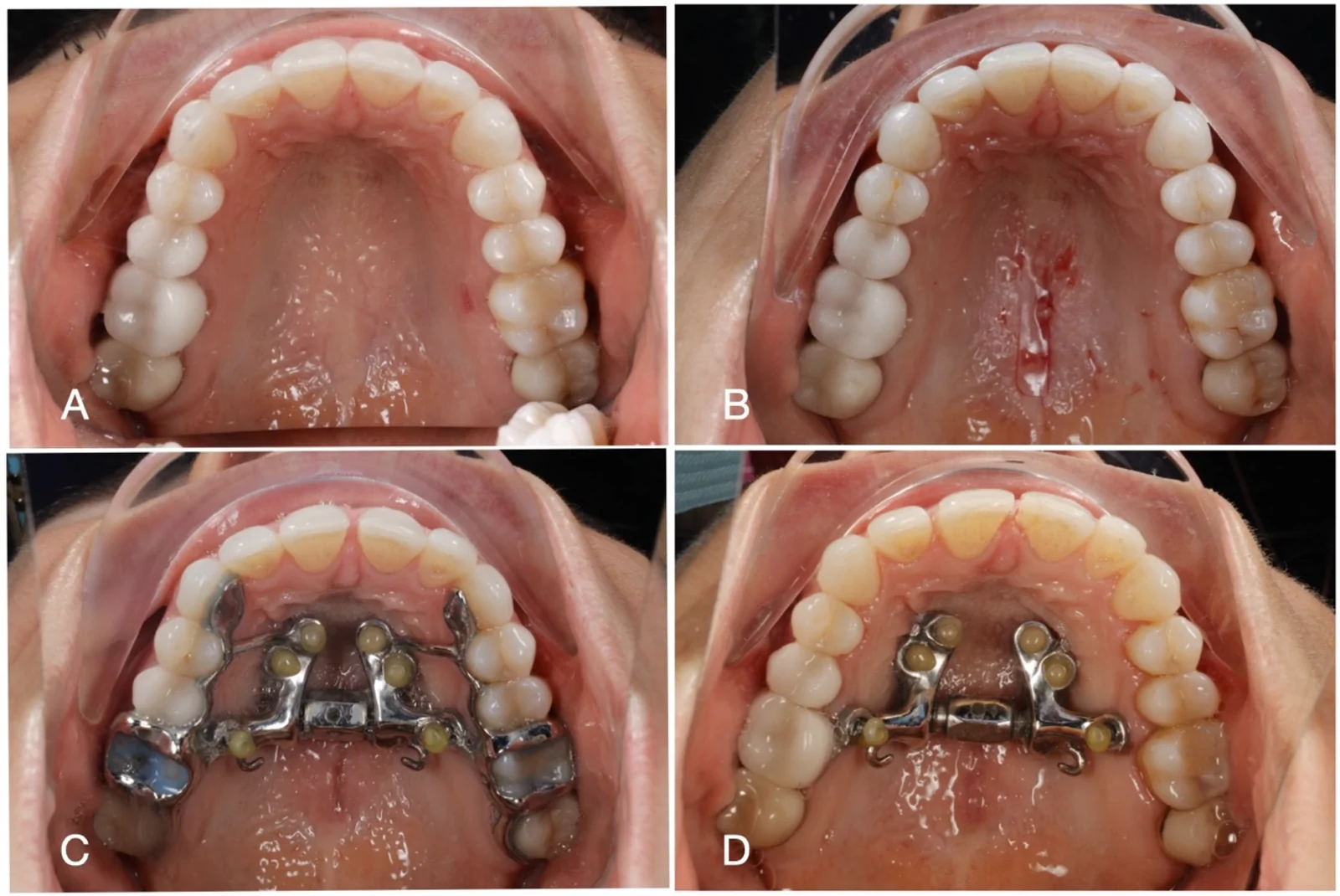
Occlusal view of the maxillary arch following the sequence of 3D-guided midpalatal piezocorticotomy- assisted mARPE expansion: **(A)** before intervention; **(B)** immediately after 3D-guide midpalatal piezocorticotomy, **(C)** custom mARPE installed, **(D)** after the completion of midfacial expansion with framework arms removed.

The custom MARPE appliance was allowed to remain passive for a one-week healing period following installation and piezocorticotomy. Following the 3D-guided piezocorticotomy procedure, a 7-day passive latency phase was observed prior to the initiation of appliance activation. This 1-week healing period is clinically essential to permit adequate mucosal and soft-tissue closure over the intentional surgical sites. Ensuring primary soft-tissue healing mitigates the risk of mucosal tearing during early expansion and prevents potential adverse events, such as oral-nasal fluid regurgitation, while still allowing the subsequent activation protocol to fully capitalize on the regional acceleratory phenomenon. Maxillary expansion was initiated exactly seven days post-operatively using an initial activation protocol of two turns per day (representing a total of 0.22 mm of linear activation daily) for the subsequent three weeks, or until clinically visible midpalatal suture separation occurred. Following clinical verification of midpalatal separation by the treating clinician, the expansion protocol was transitioned to a moderate phase of one turn per day (0.11 mm daily) for the next three weeks. Finally, the rate was decelerated to a maintenance phase of one turn every three days for the final three weeks, or until the prescribed volume of skeletal expansion was successfully achieved. (Figure 2).

### Participants

To maximize statistical power given the exploratory, pilot nature of this study and the low incidence of case events (*n* = 15), an approximate 1:4 case-to-control matching ratio was employed. Controls (*n* = 65) were selected from the same baseline patient pool and matched to cases based on demographic parity, including age profile (*p* = .29) and similar sagittal jaw relationships (*p* = 0.13). Subjects were consecutively screened and enrolled in this retrospective study based on strict clinical, diagnostic, and age-related parameters. To be eligible for inclusion, patients were required to be skeletally mature adults, defined as individuals aged 19 years or older. The inclusion criteria encompassed patients presenting with sleep-disordered breathing who concurrently demonstrated objective transverse skeletal deficiencies. Qualification for custom MARPE therapy was strictly based on reduced structural dimensions of the nasal cavity base and maxillary base as measured on baseline CBCT scans, rather than the clinical presentation of airway symptoms alone or the presence of a traditional uni- or bilateral dental crossbite. Conversely, patients were systematically excluded from the study cohort if their medical records indicated a documented history of corrective orthognathic or maxillofacial interventions, specifically including bimaxillary surgery, bilateral sagittal split osteotomy, or Le Fort I osteotomy. Additionally, candidates were excluded if they presented with systemic connective tissue disorders, active autoimmune diseases, or pre-existing temporomandibular joint dysfunction or disorders. Patients presenting without clinically evident crossbites were eligible for enrollment based on objective skeletal dimensions of the nasal base width derived from pre-treatment cone-beam computed tomography (CBCT) imaging, utilizing a strict minimum eligibility threshold of 18 mm and a maximum permissible width of 29 mm.

## Variables

### Predictor (independent) variables

The primary independent variable of interest was the pre-treatment palatal plane inclination relative to the cranial base (SN-PP_b, measured in degrees). Secondary continuous predictors included age (years), sagittal jaw relationship before treatment (ANB_b, degrees), mean midpalatal suture separation achieved through treatment (MeanSEP, mm), mean separation of the frontonasal suture (FrontoNasSEP, mm), and the occlusal plane inclination relative to the cranial base before treatment (OcP-SN_b, degrees).

### Covariate and demographic control

Biological sex was treated as a nominal, dichotomous variable (Male vs. Female, with Female serving as the reference cohort in multivariable modeling). Age was evaluated as a continuous linear variable.

### Outcome (dependent) variable

The primary dependent variable was final case-control status. Patients were categorized into case and control groups based on the degree of post-expansion skeletal downward maxillary rotation. This rotation was quantified by measuring the angle formed between the Sella-Nasion (S-N) reference plane and the palatal plane (ANS-PNS). Participants were classified into study groups using a binary outcome based strictly on absolute cephalometric parameters obtained after the expansion intervention. The ‘Case’ group (Group 1, *n* = 15) comprised individuals presenting with a post-expansion S-N/ANS-PNS angle more or equal to 11 degrees, indicating a post-treatment state of downward maxillary rotation. The ‘Control’ group (Group 2, *n* = 65) consisted of individuals whose absolute post-expansion angle remained < 11 degrees.

### Data sources

All three-dimensional hard- and soft-tissue measurements were derived from high-resolution cone-beam computed tomography (CBCT) datasets captured immediately before and after the maxillary expansion stage. To maintain geometric and voxel-level consistency, all craniofacial imaging was executed using a single Planmeca Viso G7 unit (Planmeca, Helsinki, Finland) configured to the following standardized exposure parameters: a 24.1 × 20 cm field of view (FOV), a 0.450 mm isotropic voxel size, 110 kV, 5.6 mA, and a total exposure duration of 3.125 seconds.

**Figure 3:**
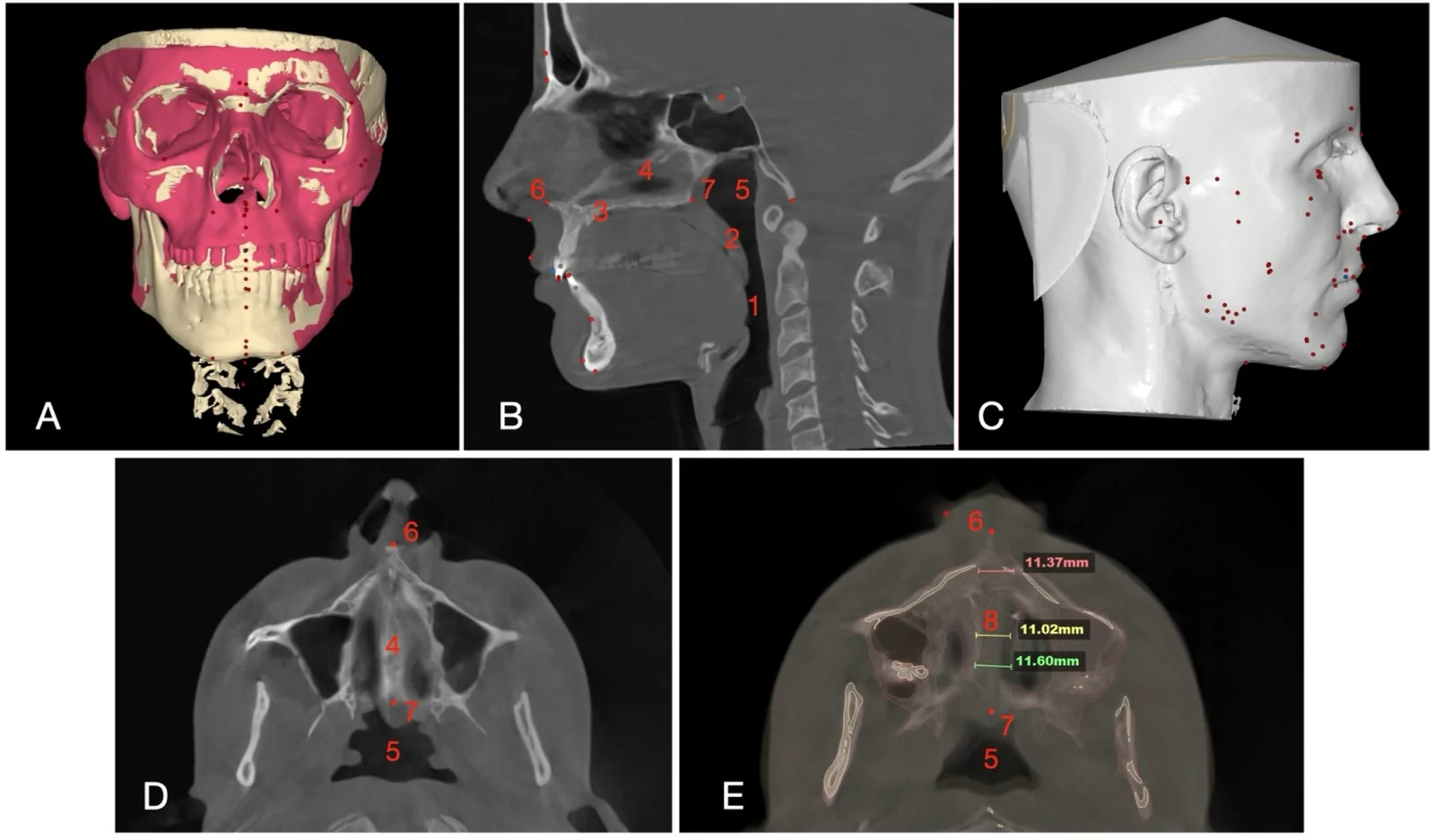
3-D cBCT measurements: **(A)** superimposition of the 3D cBCT volumes from before (beige) expansion and after the completion of expansion (magenta), **(B)** landmark allocation [1 – oropharyngeal airway space, 2 – soft palate and uvula, 3 – palatal plane, 4 – nasal septum sagittal view, 5 – nasopharyngeal airway space, 6 – anterior nasla spine (ANS), 7 – posterior nasal spine (PNS)], **(C)** soft tissue mesh landmarks, **(D)** axial view of the palatal plane with the nasal septum base before expansion, **(E)** axial view of the palatal view after completion of expansion with the vomer (8) as a reference of the nasal septum and three measured intermaxillary width measurements (anterior, medial, and posterior).

Following acquisition, the raw Digital Imaging and Communications in Medicine (DICOM) files were exported into NemoFab virtual orthognathic software, version 2026 (Nemotec, Madrid, Spain) for volumetric orientation, landmark identification, superimposition, and definitive 3-D cephalometric analysis. (Figure 3) Digital dental models and soft-tissue surface contours were generated via high-precision intraoral surface scans captured using an iTero element scanner (Align Technology, San Jose, CA, USA). These intraoral datasets were subsequently converted to stereolithography (STL) formats and digitally fused with the baseline and post-expansion CBCT coordinate spaces within the NemoFab environment to facilitate precise 3D treatment planning and surgical guide fabrication.

### Measurements

To evaluate the reproducibility of the cephalometric tracing protocol and quantify potential measurement error, an intra-examiner reliability assessment was performed. Twenty DICOMs (25% of the total sample) were randomly selected using a computer-generated sequence two weeks following the initial primary analysis. These duplicate volumes were completely de-identified, re-randomized, and re-traced by the original primary investigator, who remained strictly blinded to both the baseline clinical data and the prior landmark coordinates. The intra-examiner ICC for the primary skeletal variables demonstrated excellent reproducibility, yielding a point estimate of 0.941 with a highly precise 95% confidence interval spanning from 0.912 to 0.968 [F_(19, 19)_ = 32.84, *p* < .0001].

### Bias

Given the observational nature of this investigation, several potential sources of bias were proactively considered and minimized. First, the retrospective design introduces susceptibility to selection and information bias; however, this was mitigated by utilizing consecutive patient enrollment from a centralized database with strict, pre-defined eligibility criteria. Second, the unbalanced 1:4 case-to-control ratio (*n* = 15 vs. *n* = 65) reflects the low native incidence of case events in the clinical population. While this distribution presents an inherent demographic asymmetry, it was leveraged by design to maximize statistical power for the continuous biomarker comparisons, as confirmed by our *post-hoc* power metrics. Third, to eliminate investigator and measurement bias during radiographic interpretation, all pre-treatment CBCT landmark tracings were executed by a single operator who was completely blinded to final patient clinical outcomes and group allocations, with measurement stability mathematically verified through duplicate intra-examiner reliability testing.

### Sample size justification

A *post-hoc* power analysis was performed to evaluate the adequacy of the pilot sample size (*n* = 80; n_1 = 65 controls, n_2 = 15 cases). Based on the observed baseline distributions of the primary SN-PP_b biomarker (controls: 7.3 ± 3.1°; cases: 11.5 ± 2.9°), a two-sided Satterthwaite's *t*-test assuming unequal variances with a significance level of alpha = .05 yielded an estimated statistical power of 99.7%. This confirms that despite the small case cohort, the study was highly powered to detect the substantial effect size (delta = 4.2°) present in this population.

### Statistical methods

Statistical analyses were performed using Stata software, version StataNow/SE19.5 (StataCorp, College Station, TX, USA). Continuous variables (age, linear millimeters, and angular degrees) were evaluated for normality using the Shapiro–Wilk test. Normally distributed continuous data are presented as means and standard deviations (SD), while categorical data (biological sex) are expressed as absolute frequencies and percentages (n,%).

Baseline demographic and cephalometric characteristics were compared between the case group (Group 1) and the control group (Group 2) to evaluate cohort parity. Inter-group differences for continuous variables were evaluated using an independent two-sample t-test.

To evaluate the predictive capability of the pre-treatment craniofacial metrics, univariable (crude) logistic regression analyses were executed for each continuous and categorical variable to calculate Crude Odds Ratios (OR) with corresponding 95% confidence intervals (CI). Due to the exploratory pilot nature of the study and the limited number of case events (*n* = 15), a parsimonious multivariable logistic regression model was constructed to determine the independent effect of the primary continuous biomarker (SN-PP_b). To control for potential demographic imbalances without overfitting the model, the primary biomarker was adjusted specifically for biological sex to yield an Adjusted Odds Ratio (aOR). Individual variable significance within the regression models was determined using the Wald test.

**Figure 4:**
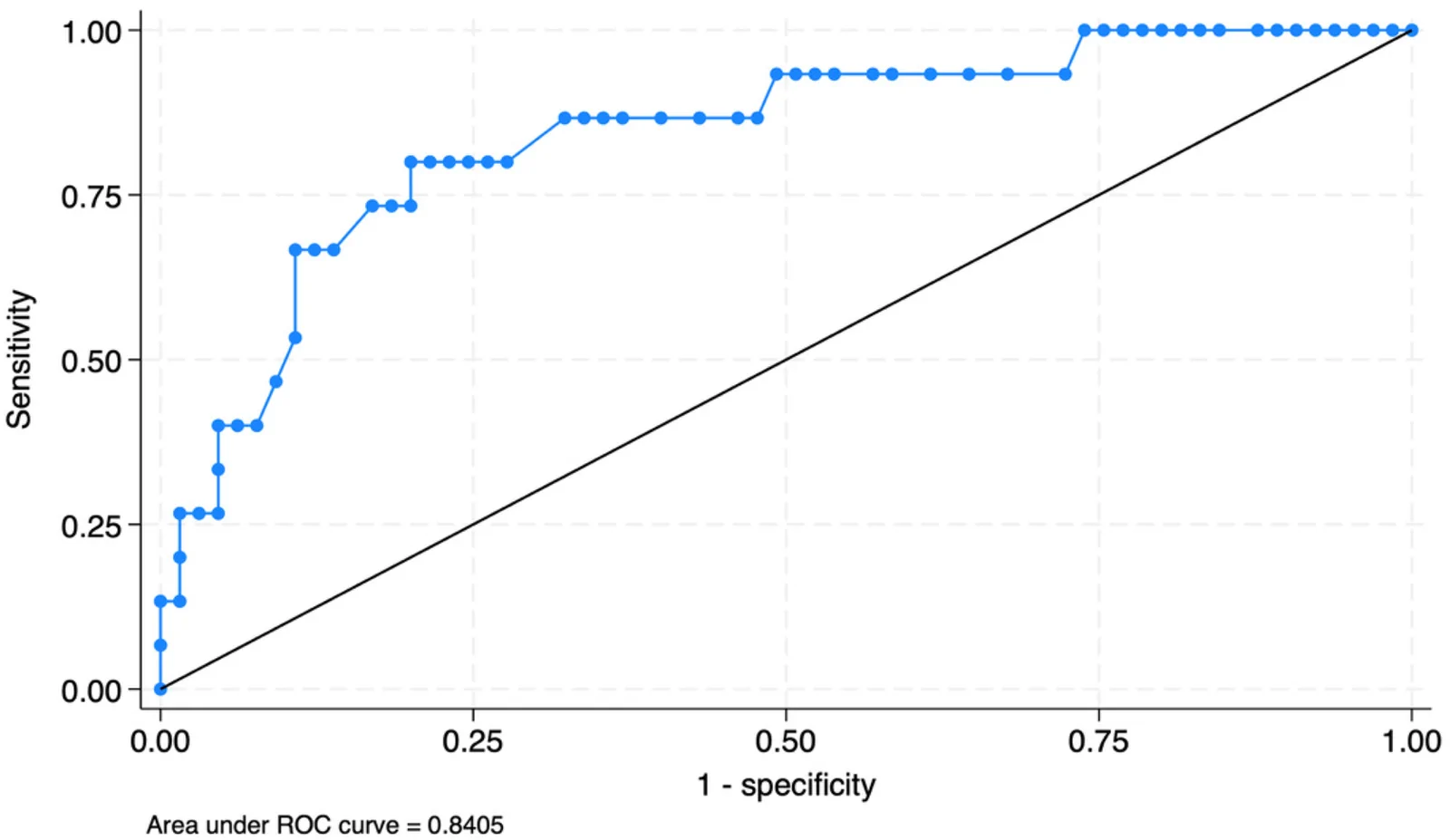
Receiver operating characteristic (ROC) curve of the pre-treatment SN-PP_b biomarker for predicting case status. The area under the curve (AUC) is 0.841 (95% CI: 0.73; 0.95). The diagonal reference line represents chance performance (AUC = 0.50).

The overall diagnostic and discriminatory performance of the pre-treatment SN-PP_b biomarker was evaluated using Receiver Operating Characteristic (ROC) curve analysis, and the Area Under the Curve (AUC) was calculated with its 95% CI (Figure 4) Intra-examiner measurement reliability for the 3D cephalometric tracing protocol was quantified using a two-way mixed-effects, absolute-agreement, single-rater intraclass correlation coefficient (ICC 1, 3) with an accompanying *F*-test. Finally, a *post-hoc* power analysis assuming unequal variances was performed using the observed baseline sample distributions of the primary biomarker to mathematically verify the adequacy of the 1:4 case-to-control sample size allocation. For all statistical tests, two-sided p-values < 0.05 were considered statistically significant.

## Results

Baseline demographic and cephalometric characteristics of the study cohort are presented in Table 1. There were no statistically significant differences between Group 1 (controls, *n* = 65) and Group 2 (cases, *n* = 15) regarding age (35.7 ± 8.4 vs. 33.1 ± 10.1 years, *p* = 0.29), baseline ANB_b angles 3.5 ± 2.7° vs. 4.6 ± 1.7°, *p* = 0.13), MeanSEP (5.9 ± 1.6 vs 5.6 ± 1.3 mm, *p* = .50), or FrontoNasSEP (1.3 ± 0.9 vs. 1.5 ± 0.9 mm, *p* = 0.46). The distribution of male subjects was 37% in the control group and 27% in the case group.

**Table 1:** Descriptive characteristics across two groups **[c**ontinuous variables are presented as mea*n* ± standard deviation (SD), and categorical variables are presented as (%), * indicates statistical significance measured at *p* < 0.05].

	Group 1, “cases” (*n* = 15) (SN-PP_a>=11°)	Group 2, “controls” (*n* = 65) (SN-PP_a < 11°)	*p-*value
*Demographic variables*			
Male sex, (%)	27	37	
Age (years ± SD)	33.1 ± 10.135	35.7 ± 8.4	0.29
*Cephalometric variables*			
ANB_b (degrees ± SD)	4.6 ± 1.7	3.5 ± 2.7	0.13
MeanSEP (mm ± SD)	5.6 ± 1.3	5.9 ± 1.6	0.50
FrontoNasSEP (mm ± SD)	1.5 ± 0.9	1.3 ± 0.9	0.46
SN-PP_b (degrees ± SD)	11.5 ± 2.9	7.3 ± 3.1	0.001^a^
SN-PP_a (degrees ± SD)	13.08 ± 1.24	7.15 ± 2.63	<0.001^a^
*Difference in SN-PP by group* Mean ± SD (*p-value)*	1.57 ± 2.83 *(0.05)*	−0.17 ± 2.87 *(0.63)*	

a Statistically significant difference between groups measured with Student t-test.

Conversely, highly significant skeletal differences were observed. The primary biomarker, SN-PP_b, was significantly steeper in cases compared to controls (11.5 ± 2.9° vs. 7.3 ± 3.1°, *p* = 0.001). Additionally, the OcP-SN_b angle was significantly greater in the case cohort (21.5 ± 5.5°) than in the control cohort (17.1 ± 5.9°, *p* = 0.01). Following the intervention, Cases demonstrated a significantly higher mean SN-PP_a of 13.08° ± 1.34° compared to Controls at 7.15° ± 2.63° (*p* < 0.001).

The results of the univariable and multivariable logistic regression analyses are presented in Table 2. In the univariable (crude) analysis, the primary continuous biomarker, SN-PP_b, demonstrated a highly significant association with case status, with a 57% increase in the odds of being a case for every one-degree increase in the angle (Crude OR=1.57; 95% CI: 1.23, 2.00; *p* < 0.001). The only other variable demonstrating a significant univariable association was OcP-SN_b (Crude OR=1.13; 95% CI: 1.02, 1.25; *p* = 0.02). Biological sex was not significantly associated with case status in the crude analysis (Crude OR=0.62; 95% CI: 0.18, 2.17; *p* = 0.46).

**Table 2:** Univariable and multivariable association of Pre-treatment variables with outcome (for every 1-degree increase in the SN-PP_b angle, the odds of a subject having a downward maxillary plane rotation post-treatment increase by 57%). CI, confidence interval; OR, odds ratio.

	Crude odds ratio (95% CI)	*p-*value	Adjusted odds ratio (95% CI)	*p-*value
SN-PP_b (per degree increase)	1.57 (1.23; 2.00)	<0.001^a^	1.51 (1.18; 1.94)	0.001^a^
Age (per year increase)	0.96 (0.90; 1.03)	0.29	-	-
Male sex (Male vs Female)	0.62 (0.18; 2.17)	0.46	-	-
ANB_b (per degree of increase)	1.19 (0.95; 1.50)	0.14	-	-
MeanSEP (per mm of increase)	0.88 (0.60; 1.28)	0.50	-	-
FrontoNasSEP (per mm of increase)	1.23 (0.71; 2.14)	0.45	-	-
OcP-SN_b (per degree of increase)	1.13 (1.02; 1.25)	0.02^a^	-	-

a *p*-values indicate statistical significance at the *p* < 0.05 threshold. Statistical significance for all univariable and multivariable odds ratios was evaluated using the Wald test derived from logistic regression models.

To evaluate the independence of the primary biomarker while respecting the sample size constraints (*n* = 15 cases), a multivariable logistic regression model was constructed adjusting for biological sex. In this adjusted model, biological sex did not act as a significant independent confounder. The adjusted odds ratio for SN-PP_b remained highly stable and robustly significant, showing a 51% independent increase in the odds of case status per degree increase (Adjusted OR=1.51; 95% CI: 1.18, 1.94; *p* = 0.001).

Receiver operating characteristic (ROC) curve analysis was performed to evaluate the diagnostic capability of the pre-treatment SN-PP_b biomarker. The area under the curve (AUC) was 0.841 (95% CI: 0.724–0.958), demonstrating excellent discrimination between cases and controls.

## Discussion

### Key results

The primary finding of this exploratory study is that individuals who exhibit a high-angle post-treatment morphology (Group 2) exhibit a distinct baseline craniofacial morphology characterized by a steeper palatal plane (SN-PP_b: 11.5 ± 2.9°) and a steeper occlusal plane (OcP-SN_b: 21.5 ± 5.5°) relative to the anterior cranial base.

Structurally, the parallel increase in both maxillo-mandibular planes points toward a coordinated downward and backward rotational pattern of the visceral cranium in cases. Because sagittal discrepancies (ANB_b) and the amount of suture separation (MeanSEP, FrontoNasSEP) were identical between the groups, this skeletal phenotype represents an isolated vertical risk metric rather than a generalized facial disharmony.

From a methodological standpoint, baseline demographics revealed a higher proportion of female subjects within the case group (73%) compared to controls (63%). This sex imbalance highlights the distinct possibility of biological sex acting as a baseline confounder. Crucially, our multivariable logistic regression addressed this skew; even when controlling for this sex distribution, the primary SN-PP_b biomarker retained a powerful independent predictive signal (aOR=1.51, *p* = .001) alongside an excellent diagnostic accuracy (AUC = 0.841). This confirms that the steeper palatal plane angle is a true structural biomarker for determining final post-treatment palatal plane status and holds clinical utility as a screening metric to identify patients predisposed to a high-angle post-treatment skeletal relationship.

Crucially, the novel pre-treatment biomarker evaluated in this pilot study demonstrated excellent diagnostic performance, yielding an AUC of 0.841. While the small sample size (*n* = 15 cases) necessitates cautious interpretation, this strong discriminatory signal suggests that the SN-PP_b angle holds clinical utility as a predictive screening metric. These promising preliminary data directly justify a larger, fully powered prospective validation trial to confirm the biomarker's definitive diagnostic thresholds.

The mechanisms of maxillary, mandibular base, and occlusal plane responses differ between various maxillary expansion techniques. The latter are widely described in the literature and include Rapid Palatal Expansion (RPE), Surgically-Assisted Rapid Palatal Expansion (SARPE), and Miniscrew-Assisted Rapid Palatal Expansion (MARPE).

Some studies involving RPE in mixed dentition reported both forward maxillary displacement and downward maxillary base rotation (13, 14), others report significant anterior facial height increase along with maxillary downward rotation (15, 16). Similarly, Chung (17) described significant downward maxillary rotation with the increase in the anterior face height following RPE is adolescents.

SARPE treatment routinely involved the use of fixed hyrax-type expanders. Studies analyzed in a systematic review and meta-analysis published by Lin and coauthors (18) demonstrated downward maxillary plane rotation when measured with SN-PP angle. On the opposite side, maxillary rotation following SARPE was described by Farfel (19) who did not report any rotation of the ANS-PNS plane.

Parhiz (5) suggested predictive parameters that were associated with the downward rotation of the maxillary plane following SARPEThose parameters included pre-existing downward rotation of the maxillary plane (measured as SN-ANS-PNS angle), gender, and age. These were shown to define 75% of the post-treatment downward rotation of the maxillary plane. Most studies on SARPE report downward rotation of the maxillary plane similar to studies on RPE. RPE was also reported to produce more dento-alveolar effects associated with molar mesial movement, molar extrusion, maxillary incisor proclination and others (7). Crucially, while a broad consensus exists regarding the prevalence of this downward maxillary displacement across expansion modalities, the specific magnitude and nature of these reported changes may be heavily influenced by variations in study reporting methods, the choice of reference planes used for patient classification, and the concurrent severity of these localized dentoalveolar effects.. While these studies directly quantified the degree of active downward rotation, our findings complement this by demonstrating that a steeper baseline morphology is highly predictive of an absolute high-angle outcome (SN-PP >= 11) following expansion.

### Limitations

A primary limitation of this exploratory study is its restricted sample size, particularly within the affected cohort; while the total sample size consisted of 80 participants, only 15 individuals represented true “cases.” This small case volume necessitates a cautious interpretation of our findings, as reduced sample sizes can limit statistical power and increase the margin of error when establishing definitive diagnostic thresholds. Furthermore, as an exploratory pilot study, these preliminary data serve primarily to identify trends rather than establish universal clinical guidelines, highlighting the distinct need for a larger, fully powered prospective trial to validate our findings.

Additionally, because our study population was drawn from a single private practice and treated using a uniform, specific protocol, the generalizability of these findings may be limited. The acute vertical skeletal changes and the predictive value of SN-PP_b identified here may vary across different clinical settings, alternative MARPE appliance designs, and diverse patient demographics. Future multicenter studies involving varied clinical protocols are necessary to confirm the broader generalizability of these outcomes.

Moreover, from a methodological standpoint, we observed a baseline demographic imbalance, with a higher proportion of female subjects within the case group (73%) compared to the controls (63%). Although our multivariable logistic regression successfully controlled for this skew—demonstrating that the primary SN-PP_b biomarker retained a powerful, independent predictive signal—the initial sex imbalance remains a baseline confounder that should be more evenly balanced in future prospective designs.

A limitation of this study is that post-treatment CBCT scans were obtained immediately following the completion of expansion, thus reflecting acute treatment effects rather than long-term post-retention stability. However, capturing the immediate post-expansion phase was a deliberate methodological choice. The immediate skeletal and rotational changes following MARPE intervention—particularly the ‘drop-down effect’ of the midface, posterior mandibular rotation, and subsequent Class II skeletal tendencies—represent a major challenge for clinicians that is difficult to reverse with orthodontics alone. Consequently, establishing an understanding of the immediate nature of this unwanted condition, and the factors predisposing patients to it at its peak presentation, is clinically paramount. Future prospective studies with long-term follow-up are required to evaluate how these immediate changes adapt over an extended retention period.

Finally, because the ROC analysis was performed on the same derivation dataset without internal or external validation, the reported diagnostic performance of SN-PP_b may be overly optimistic, necessitating future multicenter validation studies.

## Interpretation

This exploratory study demonstrates that individuals who develop the condition exhibit a distinct baseline craniofacial morphology characterized by a coordinated downward and backward rotation of the visceral cranium, specifically presenting as steeper palatal (SN-PP_b) and occlusal (OcP-SN_b) plane angles. Because sagittal relationships and suture separations remained identical between groups, this structural divergence is interpreted as an isolated, hard-tissue vertical risk metric rather than a generalized facial disharmony. Statistically, the primary SN-PP_b biomarker proved to be a highly robust and stable predictor; even when controlling for a prominent baseline sex imbalance, it maintained an excellent diagnostic accuracy (AUC = 0.841, *p* = .001) independent of patient demographics. Ultimately, these findings suggest that the palatal plane angle holds strong clinical utility as a sex-independent, pre-treatment screening metric, providing a strong justification for future large-scale prospective trials to validate definitive diagnostic thresholds.

## Generalizability

Because this investigation was conducted as an exploratory pilot study with a limited cohort size (*n* = 80 total, *n* = 15 cases), the generalizability of these findings to the broader clinical population is currently limited. Furthermore, the distinct baseline demographic skew toward female subjects means that while the SN-PP_b biomarker proved statistically robust, these preliminary thresholds cannot yet be universally applied across diverse, evenly balanced populations. Consequently, these results must be interpreted as a foundational proof-of-concept rather than an absolute clinical standard. Ultimately, this narrow generalizability highlights the distinct need for a large-scale, multi-center prospective trial to validate these diagnostic metrics on a wider scale.

## Conclusions

In conclusion, our preliminary findings suggest that a steeper pre-treatment palatal plane inclination (SN-PP) serves as a distinct, sex-independent structural biomarker that helps predict a high-angle post-treatment maxillary morphology (SN-PP >=11 degrees) in adult patients undergoing 3D-guided piezocoticotomy-assisted MARPE expansion. Individuals presenting with a steeper baseline palatal plane display a heightened likelihood of exhibiting this final hyperdivergent relationship and its associated vertical characteristics post-expansion. While our localized diagnostic performance model shows strong clinical promise, these results are preliminary and stem from a single-center retrospective pilot cohort. Ultimately, these findings underscore the potential utility of SN-PP thresholds for initial clinical risk stratification, though larger prospective multi-center studies are required to establish its definitive role in treatment planning.

## Data Availability

The raw data supporting the conclusions of this article will be made available by the authors, without undue reservation.
